# Morphological features of the inferior fascicle of the anterior inferior tibiofibular ligament

**DOI:** 10.1038/s41598-019-46973-4

**Published:** 2019-07-18

**Authors:** M. Edama, M. Takeishi, S. Kurata, T. Kikumoto, T. Takabayashi, R. Hirabayashi, T. Inai, M. Ikezu, F. Kaneko, I. Kageyama

**Affiliations:** 1 Institute for Human Movement and Medical Sciences, Niigata University of Health and Welfare, Niigata, Japan; 2 Department of Anatomy, School of Life Dentistry at Niigata, Nippon Dental University, Niigata, Japan

**Keywords:** Anatomy, Ligaments

## Abstract

In this study, the inferior fascicle of the anterior inferior tibiofibular ligament (AITFL) was classified to provide basic information to help elucidate the mechanism of ankle joint anterolateral impingement, and the morphological features of each type were compared for the purpose of clarification. This investigation examined 100 feet from 52 cadavers. The AITFL was classified into four types according to the presence or absence of the inferior fascicle and the positional relationship between the AITFL and the inferior fascicle of the AITFL. The morphological features of the AITFL that were measured included the fibre bundle length, fibre bundle width, fibre bundle angle, and the distance between the joint levels. A distinct, independent inferior fascicle of the AITFL was identified in 15 feet (15%). There were no significant differences in the morphological features based on differences in the AITFL classification. Therefore, these findings suggest that the presence or absence of the inferior fascicle and the difference in the positional relationship between the AITFL and the inferior fascicle of the AITFL are less likely to be involved in impingement during ankle dorsiflexion.

## Introduction

Ankle impingement syndromes are painful conditions caused by friction of joint tissues. The leading causes of impingement lesions are traumatic ankle injuries, usually ankle sprains, which result in chronic ankle pain^1,2^. Impingement syndromes of the ankle involve either osseous or soft tissue impingement and can be anterior, anterolateral, or posterior^2,3^. Three different types of intra-articular soft tissue pathologies, including hypertrophied synovium, meniscoid lesions, and an impinging inferior fascicle of the anterior inferior tibiofibular ligament (AITFL), have been reported to cause chronic ankle pain after an inversion injury^2,4–11^. Among them, discussion about impingement by the inferior fascicle of the AITFL continues^12–14^.

Bassett *et al*.^12^ found that the inferior fascicle of the AITFL was present in 10 of 11 feet in a study using fresh cadavers, and they reported that the inferior fascicle contacted the anterolateral corner of the talus at an average ankle dorsiflexion angle of 12° (9°–17°). In addition, they treated 7 patients who had talar impingement by an AITFL, and the patients were followed for a mean of 39 months. Two patients had arthroscopy, and five had an arthrotomy. In all patients, a thickened inferior fascicle of the AITFL was resected. Five patients reported no pain in their ankle or limitation in activity, and the results were considered excellent. Therefore, they postulated that the posttraumatic anterolateral hyperlaxity due to an injured anterior talofibular ligament (ATFL) resulted in anterior extrusion of the talar dome with dorsiflexion, which then contacted the inferior fascicle of the AITFL with greater pressure and friction. However, in a study using fresh cadavers in a similar fashion, Akseki *et al*.^13^ found that the inferior fascicle of the AITFL was present in 39 of 47 feet. They also reported that contact between the ligament and the anterolateral talar dome was observed in 42 of 47 feet (89.3%) in the neutral position. In 37 of these 42 feet, there was a separate fascicle. Furthermore, the mean width and length of the fascicle with bending during dorsiflexion and dorsiflexion-eversion were significantly higher than without bending. Therefore, they concluded that their findings showed that the presence of the inferior fascicle of the AITFL and its contact with the talus is a normal finding, but it may become pathological due to anatomical variations and/or instability of the ankle resulting from torn lateral ligaments. In order to examine the effects of individual differences in this inferior fascicle, Ray and Kriz^14^ classified the AITFL into seven types in a study using fresh cadavers. They reported that 10 of 46 specimens (21.7%) demonstrated a separate and distinct inferior fascicle of the AITFL. Of these, 7 cadavers (70%) showed both ligamentous impingement and a bevel of the talar dome. Although a distinct inferior fascicle of the AITFL was not present in the latter types, 22 of 36 cadavers (61.6%) demonstrated varying degrees of ligamentous impingement and bevelling on the talus.

As described above, concerning the soft tissue impingement of the ankle, involvement of the inferior fascicle of the AITFL has been suggested, but a definite conclusion has not been reached. We believe that this is due to the fact that the number of cadavers targeted included as few as 11–47 feet, and that biomechanical verification and clinical research have been conducted with insufficient morphological data.

Therefore, in the present study, the inferior fascicle of the AITFL was classified by type using a large number of cadavers to provide basic information to help elucidate the mechanism of ankle joint anterolateral impingement, and the morphological features of each type (fibre bundle length, fibre bundle width, the angle of the fibre bundle to the joint, and the distance between the joint levels) were compared for the purpose of clarification.

## Methods

### Cadavers

All cadavers used in the present study were legally donated to the Nippon Dental University of Life Dentistry at Niigata in Japan. The present study was conducted in accordance with the Declaration of Helsinki. This investigation examined 100 legs from 52 Japanese cadavers (mean age at death, 79 ± 13 years; 55 sides from men, 45 sides from women; 51 sides from the right, 49 sides from the left) that had been switched to alcohol after placement in 10% formalin. None showed signs of previous major surgery around the ankle.

### Methods

In the AITFL dissection procedure, cadaver specimens of the lower legs were prepared by first cutting them off 10 cm above the ankle. The skin, subcutaneous tissue, and crural fascia were then removed, and the AITFL, ATFL, and the calcaneofibular ligament (CFL) were carefully dissected. The AITFL was then classified by morphological features of the inferior fascicle of the AITFL. The classification method was modified with reference to a previous study^14^. In this classification method, Type I is a distinct inferior fascicle that is separated from the rest of the ligament; the inferior fascicle is separated completely from the main portion of the ligament by a gap. Type II is a distinct inferior fascicle with either its proximal or its distal attachment continuous with the rest of the ligament; the gap does not completely separate the inferior fascicle from the rest of the ligament. Type III is when the lower portion of the ligament includes the inferior fascicle with the attachments to the rest of the ligament. Type IV is a ligament with no separations or gaps within its structure; it may or may not have a fascicular arrangement.

**Figure 1:**
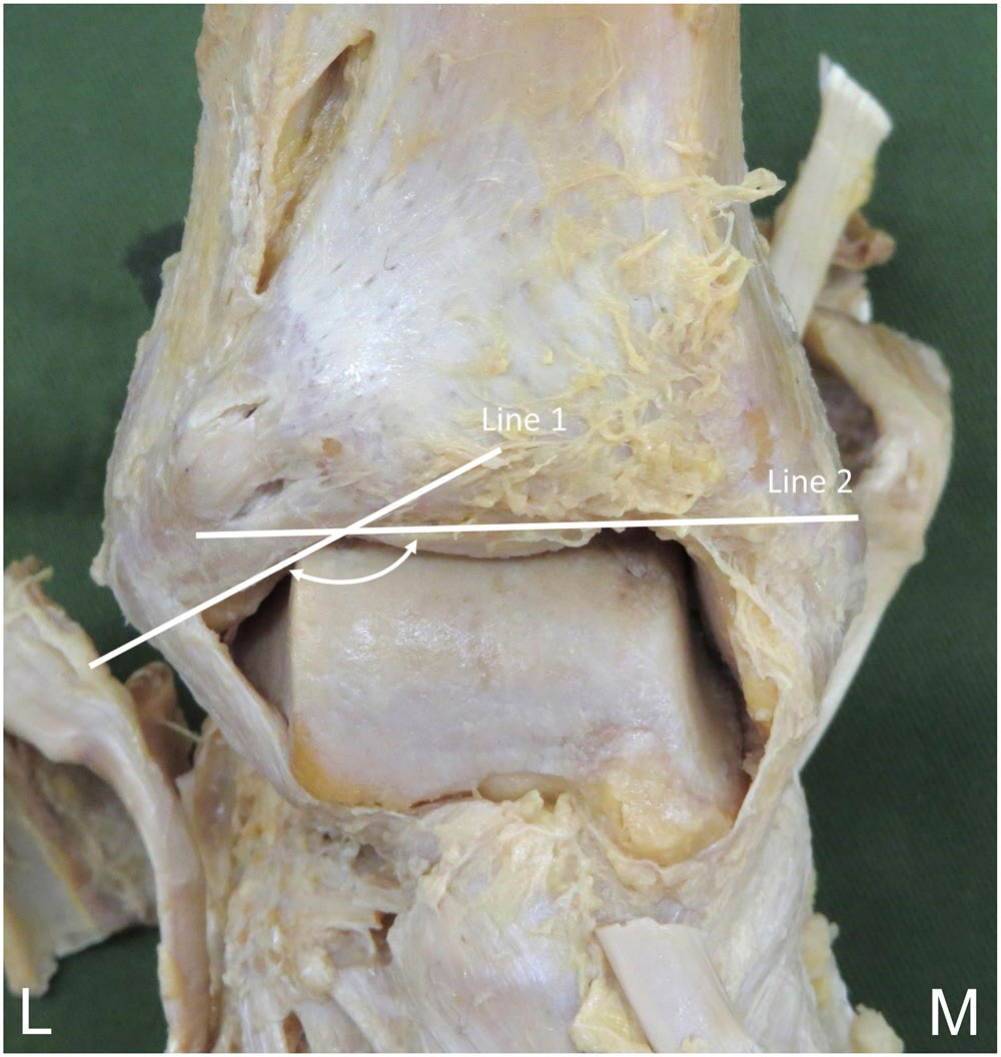
Measurement of the declination angle of the inferior fascicle of the anterior inferior tibiofibular ligament, right side, anterior. Line 1: A line parallel to the lowest inferior fascicle of the anterior inferior tibiofibular ligament. Line 2: A second line formed by connecting two points on the anterior border of the tibial plafond. White arrow: the declination angle. L: lateral, M: medial.

The morphological features of the AITFL that were measured included the fibre bundle length, fibre bundle width, the angle of the fibre bundle to the joint, and the distance between the joint levels. Fibre bundle length was measured in the middle portion of the ATFL using callipers (Digital Caliper, Shinwa, Niigata, Japan) with reference to a previous study^15^. Fibre bundle width was measured using callipers at three sites, a proximal site (fibular attachment), an intermediate site, and a distal site (talar attachment). The angle (declination angle) of the inferior fascicle of the AITFL was also measured and recorded with reference to a previous study^14^. This angle was defined as the obtuse angle formed by the intersection of the following: a line parallel to the lowest fibres of the AITFL and a second line formed by connecting two points representing the extreme medial and lateral points on the anterior border of the tibial plafond. The specimens were placed with the plantar surface on a horizontal platform, and the angle was measured using a goniometer (Goniometer, Nishikawashinwa, Tokyo, Japan) (Fig. 1). The distance between the fibular insertion and the joint level was measured between the joint level and the distal insertion point of the fascicle using callipers. All measurements were repeated three times by the same person, and the average values were recorded.

### Statistical analysis

The chi-squared test was used for comparisons between men and women and between right and left in the classification of the inferior fascicle of the AITFL. Comparisons of fibre bundle length, fibre bundle width, the declination angle, and the distance between the joint level and the insertion point within each type in the classifications of the inferior fascicle of the AITFL were made by one-way analysis of variance (ANOVA). The level of significance was taken to be 5%.

### Ethics approval and consent to participate

The methods were carried out in accordance with the 1964 Helsinki Declaration, and the cadavers were legally donated for the research by the Nippon Dental University of Life Dentistry at Niigata in Japan. Informed consent was obtained from the families of all subjects.

## Results

### Classification of the inferior fascicle of the AITFL

**Figure 2:**
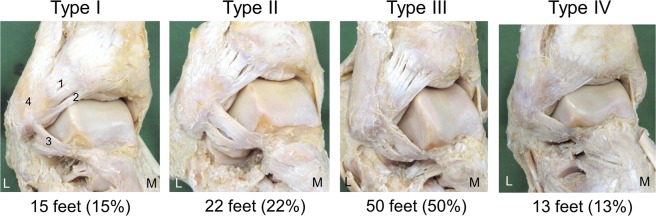
Classification of the inferior fascicle of the anterior inferior tibiofibular ligament, right side, anterolateral. 1: Anterior inferior tibiofibular ligament, 2: Inferior fascicle of the anterior inferior tibiofibular ligament, 3: Anterior talofibular ligament, 4: Fibula, L: Lateral, M: Medial. Type I: Distinct inferior fascicle is separated from the rest of the ligament. The inferior fascicle is separated completely from the main portion of the ligament by a gap. Type II: Distinct inferior fascicle with either its proximal or its distal attachment continuous with the rest of the ligament. A gap does not completely separate the inferior fascicle from the rest of the ligament. Type III: Lower portion of the ligament includes the inferior fascicle with the attachments to the rest of the ligament. Type IV: Ligament with no separations or gaps within its structure. It may or may not have a fascicular arrangement.

The inferior fascicle of the AITFL type was Type I in 15 feet (15%), Type II in 22 feet (22%), Type III in 50 feet (50%), and Type IV in 13 feet (13%) (Fig. 2). There were no significant sex-based differences, and on measuring both feet from 48 cadavers (54 legs from 27 male cadavers and 42 legs from 21 female cadavers), there were no significant differences between left and right legs (Table 1).

**Table 1:** Differences by sex and left and right sides of each type.

	Type I	Type II	Type III	Type IV	Average
Male	9 (16.4)	11 (20)	28 (50.9)	7 (12.7)	55 (100)
Female	6 (13.3)	11 (24.4)	22 (50.0)	6 (13.3)	45 (100)
Right	7 (14.6)	8 (16.7)	25 (52.1)	8 (16.6)	48 (100)
Left	7(14.6)	13 (27.1)	23 (47.9)	5 (10.4)	48 (100)

Number (%).

For examining differences between left and right legs, both legs of 48 cadavers (54 legs from 27 male cadavers and 42 legs from 21 female cadavers) were measured.

### Morphological features based on differences in the AITFL classification

No significant differences in morphological features were seen based on differences in the AITFL classification (Table 2).

**Table 2:** Measurements of the inferior fascicle of the AITFL.

Type	Length (mm)	Width (mm)			Declination angle (°)	Distance between the joint level and insertion point (mm)	Attachments of the ATFL (N)
		Proximal	Intermediate	Distal			
**I**	21.7 ± 6.0	5.0 ± 1.4	3.6 ± 0.9	3.4 ± 0.9	150 ± 6.6	16.3 ± 2.8	5/15
**II**	23.9 ± 3.1	5.0 ± 1.6	3.7 ± 1.4	3.2 ± 0.9	147 ± 5.6	14.2 ± 3.9	10/22
**III**	23.5 ± 5.3	5.2 ± 1.5	3.9 ± 1.5	3.8 ± 1.1	147 ± 17.4	15.0 ± 3.8	22/50
**IV**					148.0 ± 9.2	14.2 ± 3.3	7/13
**Mean**	23.3 ± 5.1	5.1 ± 1.4	3.8 ± 1.4	3.6 ± 1.0	147 ± 6.5	14.9 ± 3.5	
**Total**							44/100 (44%)

Values are means ± standard deviation.

Fibre bundle width was measured at three sites, a proximal site (fibular attachment), an intermediate site, and a distal site (talar attachment).

### Relationship between the AITFL and the ATFL

**Figure 3:**
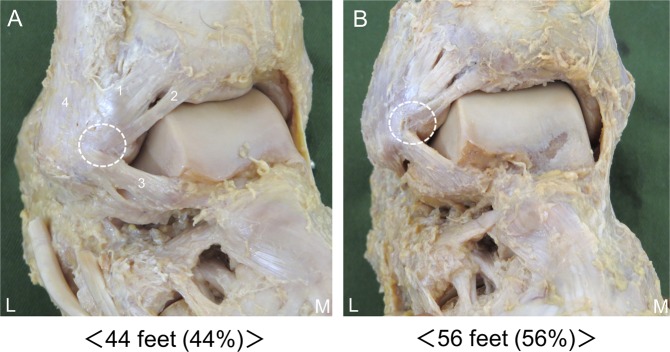
Relationship between the AITFL and the ATFL, right side, lateral. 1: Anterior inferior tibiofibular ligament, 2: Inferior fascicle of the anterior inferior tibiofibular ligament, 3: Anterior talofibular ligament, 4: Fibula. (**A**) AITFL connected to the ATFL. (**B**) AITFL not connected to the ATFL. L: Lateral, M: Medial. White circle: Relationship between the AITFL and the ATFL.

The AITFL and ATFL were connected in 44 of 100 feet (44%) (Fig. 3). No significant differences were seen among the types.

## Discussion

This study examined the morphological features (fibre bundle length, fibre bundle width, the declination angle, and the distance between the joint levels) according to the AITFL classification. To the best of our knowledge, there have been no previous large-scale anatomical studies of the morphological features of the inferior fascicle of the anterior inferior tibiofibular ligament.

In this study, a distinct inferior fascicle of the AITFL was identified in 15 feet (15%) (Type I). Previous anatomical studies reported a distinct inferior fascicle of the AITFL in 21.7% (10 of 46 feet)^14^, 82.9% (39 of 47 feet)^13^, 90.9% (10 of 11 feet)^12^, and 91.7% (22 of 24 feet)^15^, and previous MR imaging studies have reported it in 89% (32 of 36 feet)^16^; thus, the reported incidence of the inferior fascicle differs widely. One major reason for this is thought to be the very small number of specimens examined. Furthermore, the difference in classification methods may also have an effect. The results of the present research are based on examination of 100 feet, and since they were classified into four types in more detail, the result is considered highly reliable.

In the present study, no significant differences in morphological features were found based on differences in the classification of the inferior fascicle of the AITFL. Akseki *et al*.^13^ stated that wider and longer fascicles had more potential to become pathological than thinner ones. They also concluded that, if the fibular insertion point is far from the joint level, the fascicle has more potential to become pathological. In addition, Ray and Kriz^14^ reported that the angle of declination may be involved in impingement. Therefore, it was suggested that the presence or absence of the inferior fascicle and the difference in the positional relationship between the AITFL and the inferior fascicle of the AITFL are less likely to be involved in impingement during ankle dorsiflexion.

The AITFL and ATFL were connected in 44 of 100 feet (44%) at a proximal site (fibular attachment). Previous studies^17,18^ reported that the ATFL is the most important ligament in ankle stability. Bassett *et al*.^12^ postulated that the posttraumatic anterolateral hyperlaxity due to an injured ATFL resulted in anterior extrusion of the talar dome with dorsiflexion, which then contacted the inferior fascicle of the AITFL with more pressure and friction. Therefore, impingement is not only secondary to ATFL injury, but may also occur due to complex damage of the AITFL and ATFL.

The limitation of this study is that only the morphological features of the AITFL in fixed cadavers were examined. In the future, examination targeting *in vivo* samples using ultrasonography and MR imaging will be needed.

In conclusion, in this study, the inferior fascicle of the AITFL was classified, and its morphological features were clarified. It was found that there were no significant differences in morphological characteristics between the types. Therefore, the presence or absence of the inferior fascicle and the difference in the positional relationship between the AITFL and the inferior fascicle of the AITFL appear to be less likely involved in impingement during ankle dorsiflexion. Furthermore, impingement occurs not only secondary to ATFL injury, but it may also be due to complex damage of the AITFL and ATFL. Biomechanical studies using these research results as basic information are now needed.

## Data Availability

The datasets generated during and/or analysed during the current study are available from the corresponding author on reasonable request.
